# The Moral of Recent Experience

**Published:** 1921-06-04

**Authors:** 


### LUNACY AND LITIGIOUSNESS.

### The Moral of Recent Experience.

There is a form of insanity well-known to alienists of which the most prominent characteristic is the desire to appeal to authority whenever anything occurs in the patient's life which is contrary to *his* idea of the fitness of things. As he resents so much that does occur he is apt—if at large—to be almost as constantly embroiled in legal disputes as if he were one of the Jarndyce family! Often enough he retains a sufficient amount of self-control to keep out of an asylum; and, as he—or perhaps more often she—is exceedingly plausible he succeeds in enlisting much sympathy among those who do not know him well. Much of the agitation in the Press and elsewhere is fomented by such people with an imaginary grievance; and an immense amount of valuable time is constantly being wasted in this manner. Even when under care these patients are an almost constant worry to those who have charge of them. They insist on airing their imaginary grievances; and it is they who write long letters to all the duly constituted legal authorities, even to the King himself, demanding an investigation of their case.

Occasionally it happens that one of these litigious individuals, being at large, manages to enlist the aid of some legal adviser and proceeds to bring an action into court. A recent case has well exemplified the potentialities for causing annoyance, waste of time, energy, and money which such people possess. It seems a pity that in these enlightened days some way cannot be found to obviate the necessity of the whole cumbrous mechanism of the law being set in action in order to decide whether or not a doctor has acted in good faith when he signs a lunacy certificate—especially when the prime mover in the matter may be not merely an alleged lunatic, but one who has been found to be of unsound mind not only by the examining medical men, but also by visiting Commissioners. There are properly constituted authorities to whom the alleged lunatic has the right of appeal, for example, the Lord Chancellor or his visitors, the Home Secretary, or the Commissioners of the Board of Control. A lot of egregious nonsense is talked and written by people who neither know anything about these matters nor will take the trouble to ascertain the facts, as to the supineness and the incompetence of these authorities. It may be taken for a fact that any reasonable complaint of ill-treatment or malpraxis of any kind receives the attention it deserves. Surely some intermediary of this kind should be adequate as a Court of Appeal for the litigious one! In any case, it should be sufficient for him to have their decision as to whether or not an action will lie against their alleged persecutors.

An ordinary judge admits as a rule his incapacity to deal with lunacy matters of this kind, and it is obvious that he would more easily be able to come to a more valuable decision had he the assistance of assessors with an expert knowledge of lunacy. It cannot be said that a jury affords the best imaginable support to the judge in matters of this kind. The present methods are such that the medical man who has acted in good faith may yet find himself impugned for an error of judgment and mulcted in damages. Witness the verdict in the recent case of the actress who set herself on fire in an asylum and later claimed damages—which she obtained. It was a disastrous decision and one which may have far-reaching effects. If it is followed up by others of a similar character it must result in a curtailment of privileges and of liberty generally. Fortunately in the case under discussion the verdict was adverse to the plaintiff. Fortunately, because if the medical man who acts in good faith is going to be the target for any litigious lunatic who, having recovered or during a quiescent phase of his disorder, calls for and obtains legal assistance in persecuting the doctors who certified him, they will be more chary even than they are now of giving the certificates without which a dangerous or troublesome lunatic cannot be detained under care.

